# Impact of Obesity on Early In-Hospital Outcomes after Coronary Artery Bypass Grafting Surgery in Acute Coronary Syndrome: A Propensity Score Matching Analysis

**DOI:** 10.3390/jcm11226805

**Published:** 2022-11-17

**Authors:** Ihor Krasivskyi, Kaveh Eghbalzadeh, Borko Ivanov, Stephen Gerfer, Clara Großmann, Anton Sabashnikov, Elmar Kuhn, Navid Mader, Ilija Djordjevic, Thorsten Wahlers

**Affiliations:** 1 Department of Cardiothoracic Surgery, University Hospital Cologne, 50937 Cologne, Germany; 2 Department of Cardiothoracic Surgery, Helios Hospital Siegburg, 53721 Siegburg, Germany

**Keywords:** obesity, BMI, CABG, mortality

## Abstract

Recent advances in perioperative care have considerably improved outcomes after coronary artery bypass graft (CABG) surgery. However, obesity can increase postoperative complication rates and can lead to increased morbidity and mortality. Between June 2011 and October 2019, a total of 1375 patients with acute coronary syndrome (ACS) underwent cardiac surgery and were retrospectively analyzed. Patients were divided into 2 groups: non-obese (body mass index (BMI) < 30 kg/m^2^, *n* = 967) and obese (BMI ≥ 30 kg/m^2^, *n* = 379). Underweight patients (*n* = 29) were excluded from the analysis. To compare the unequal patient groups, a propensity score-based matching (PSM) was applied (non-obese group (*n* = 372) vs. obese group (*n* = 372)). The mean age of the mentioned groups was 67 ± 10 (non-obese group) vs. 66 ± 10 (obese group) years, *p* = 0.724. All-cause in-hospital mortality did not significantly differ between the groups before PSM (*p* = 0.566) and after PSM (*p* = 0.780). The median length of ICU (*p* = 0.306 before PSM and *p* = 0.538 after PSM) and hospital stay (*p* = 0.795 before PSM and *p* = 0.131 after PSM) was not significantly higher in the obese group compared with the non-obese group. No significant differences regarding further postoperative parameters were observed between the unadjusted and the adjusted group. Obesity does not predict increased all-cause in-hospital mortality in patients undergoing CABG procedure. Therefore, CABG is a safe procedure for overweight patients.

## 1. Introduction

The drastic increase in the overweight population worldwide is based on a permanent improvement in socioeconomic conditions and changes in dietary habits over the last years [1,2,3]. This condition affects up to 40% of the population of industrialized countries, and has led to a higher number of obese patients with concomitant comorbidities, such as peripheral vascular disease, stroke, and type 2 diabetes mellitus [4,5].

A large number of studies showed that obesity did not significantly increase postoperative complications after a bypass procedure [6,7,8,9,10,11]. However, several studies stated that the wound infection rate was significantly higher in obese patients as compared to non-obese patients [12,13]. Moreover, additional authors demonstrated that morbid obesity (BMI ≥ 40 kg/m^2^) seems to be an independent predictor of increased mortality after CABG surgery [6]. Other studies have also showed that obesity could affect the short and long-term outcomes of patients undergoing bypass procedure [13,14,15,16,17].

Consistent progress in operative skills and perioperative care considerably improved the outcomes after CABG surgery. However, older patients with comorbidities, such as obesity and diabetes, can increase postoperative complication rates and lead to increased morbidity and mortality [6,11,12,18,19,20].

The association between bypass surgery in acute coronary syndrome (ACS) and obesity-related outcomes still remains unclear. In this study, we aimed to investigate the impact of obesity on early in-hospital outcomes after coronary artery bypass grafting surgery in acute coronary syndrome.

## 2. Materials and Methods

The study was designed as a retrospective single center, non-randomized analysis of our CABG cohort. From June 2011 until October 2019, a total of 1375 patients underwent bypass surgery for ACS at the University Hospital Cologne. In order to analyze weight-associated differences in early clinical outcomes, patients were divided into weight categories according to BMI. 

Our patients were divided into 6 categories according to World Health Organization (WHO) definition of obesity:

- *Underweight: BMI < 18.5 kg/m^2^*
- *Normal weight BMI 18.5–24.9 kg/m^2^*
- *Pre-obesity BMI 25.0–29.9 kg/m^2^*
- *Obese class I BMI 30.0–34.9 kg/m^2^*
- *Obese class II BMI 35.0–39.9 kg/m^2^*
- *Obese class III BMI > 40.0 kg/m^2^*

**Figure 1:**
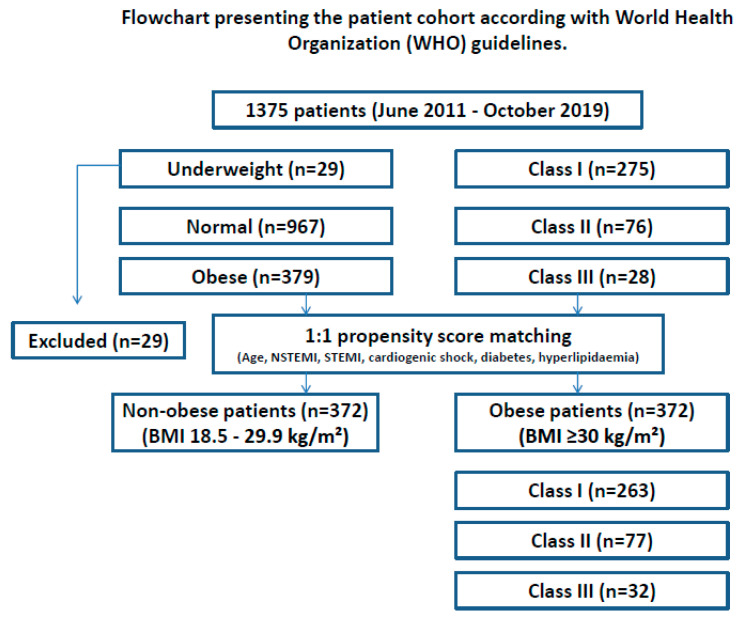
Flowchart presenting the patient cohort according to the World Health Organization (WHO) guidelines before and after PSM. BMI—body mass index, PSM—propensity score matching, n—number of patients.

All relevant data (demographic, clinical, preoperative, operative, and early postoperative data) were analyzed retrospectively after extraction from our institutional database. To compare the unequal patient groups, a propensity score-based matching (PSM) was applied as described below (Figure 1). 

To analyze the influence of obesity on clinical outcomes, patients were divided into 2 groups: non-obese (BMI 18.5–29.9 kg/m^2^, *n* = 967), and obese (BMI ≥ 30 kg/m^2^, *n* = 379). Underweight patients (*n* = 29) were excluded from the analysis. Furthermore, patients were divided into 3 obesity classes. BMI associated all-cause in-hospital survival and all relevant clinical outcomes were compared between the two groups.

### 2.1. Surgical Approach

All cases were performed through a median sternotomy. Surgical techniques were standardized according to the preferences of each surgeon. CPB was established between the ascending aorta and either the right atrium using a 2-stage cannula or cannulation of both venae cavae. Myocardial protection was achieved by using Buckberg or Calafiore cardioplegia in an antegrade and/or retrograde technique. CABG procedures were performed in patients with coronary artery disease. According to a previous risk scoring, the left internal mammary artery (LIMA) and the right internal mammary artery (RIMA), and/or the great saphenous vein (SVG), were harvested for venous grafts. An activated clotting time (ACT) with a mean value of 450s was achieved with intravenous heparin. All anastomoses were performed with a 7-0 or 8-0 monofilament suture. The side-biting clamp technique was used to perform the proximal anastomosis. In cases of combined valve and CABG surgery, coronary anastomoses were performed first. After aortotomy, the diseased aortic valve was excised and the annular calcium was debrided. The annulus was measured with the dedicated sizers. Valve function was reviewed by transoesophageal echocardiography during the operation. Temporary epicardial pacing was utilized according to the surgeon’s preference or need during dislocation of the heart to the appropriate target vessel. All patients received anticoagulation according to the standard protocol using in our heart institution.

### 2.2. Data Collection

Clinical, pre-operative, operative and post-operative data of all patients was extracted from a computerized database based on the mandatory German Cardiac Surgery Quality Assurance System. Data were collected during the patients’ hospital stays and analyzed retrospectively. All patients were analyzed with respect to relevant perioperative data and in-hospital survival.

### 2.3. Outcome Analysis

The primary outcome in our study was all-cause in-hospital mortality after CABG-surgery. Secondary outcomes were: perioperative myocardial infarction (cardiac symptoms, electrocardiography (ECG) changes, or imaging findings), renal failure requiring dialysis (glomerular filtration rate (GFR) < 15 mL/min, life-threatening hyperkalemia, refractory acidosis and hypervolemia causing end-organ complications), respiratory failure (PaO_2_/FiO_2_ ≤ 100 mmHg with PEEP ≥ 5 cm H_2_O, re-intubation or prolonged weaning), bleeding requiring re-operation (blood loss with a hemoglobin decrease of greater than 3 g/dL, any hemoglobin decrease of greater than 4 g/dL or transfusion of 2 units blood products or more), stroke (ischemic stroke or hemorrhagic stroke) and length of intensive care unit (ICU) and hospital stay.

### 2.4. Ethics

The study was conducted in accordance with the Declaration of Helsinki (as revised in 2013). The manuscript was submitted to the local Ethics Committee of the Medical Faculty of the University of Cologne. They stated that we are exempt from applying for ethical approval as under German law; no separate ethics application or statement of ethical approval by the local ethics committee are required for performing purely retrospective clinical studies.

### 2.5. Statistical Methods

Statistics was performed using Student *t*-test or Mann-Whitney-U test, each depending whether continuous variables are normally distributed or not. The Chi-square test was used for categorical variables. Continuous variables are expressed as mean ± standard deviation (SD). As long as the data for the ICU and in-hospital stay were not normally distributed, the median was presented. Categorical variables are presented as percentage of the sample. The Fisher exact test was performed when the minimum expected count of cells was <5. Logistical regression was conducted in order to create the predicted variable. A rigorous 1:1 nearest neighbor-matching algorithm without replacement was used with a 0.2 caliper set. Standardized mean differences (d-values) were calculated, and absolute d-values under 0.2 were considered an indicator of adequate balance and sufficient reduction of bias. The difference between groups was tested with ANOVA. The optimal cut-off values were defined as the values that provided highest sensitivity and specificity. A *p*-value < 0.05 was considered as significant. Statistical analysis was performed using Statistical Package for Social Sciences, version 28.0 (SPSS Inc., Chicago, IL, USA).

## 3. Results

### 3.1. Baseline and Preoperative Data before and after PSM

Preoperative characteristics of the two groups (non-obese, *n* = 967; obese, *n* = 379) are shown in Table 1. After 1:1 PSM, both groups (non-obese, *n* = 372; obese, *n* = 372) were well equalized. Diabetes (*p* = 0.004) and hyperlipidemia (*p* = 0.004) were significantly higher in the non-obese group after PSM compared to the obese group. Further preoperative data did not differ between the two groups.

### 3.2. Intraoperative Data before and after PSM

Table 2 shows all relevant intraoperative data before and after PSM. No significant differences regarding intraoperative parameters were observed between the unadjusted (non-obese, *n* = 967; obese, *n* = 379) and the adjusted group (non-obese, *n* = 372; obese, *n* = 372).

### 3.3. Postoperative Data with Primary and Secondary Outcome Parameters before and after PSM

**Figure 2:**
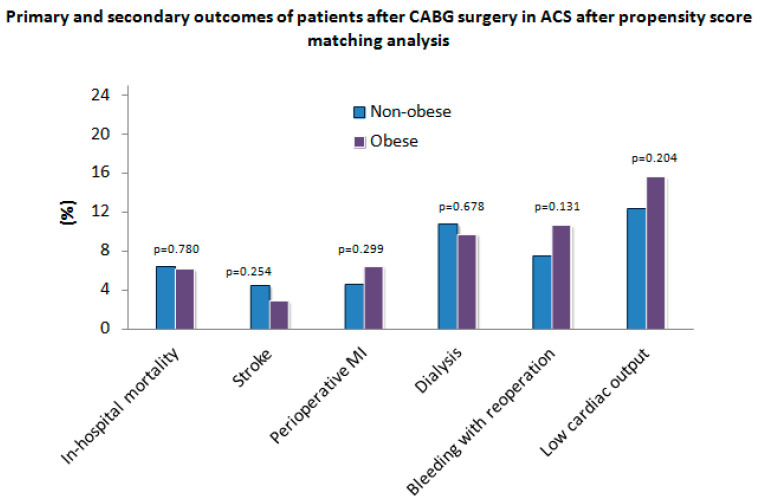
Primary and secondary outcomes of patients after coronary artery bypass grafting (CABG) surgery in acute coronary syndrome (ACS) after propensity score matching analysis. MI—myocardial infarction.

All relevant postoperative results before (non-obese, *n* = 967; obese, *n* = 379) and after (non-obese, *n* = 372; obese, *n* = 372) PSM are shown in Table 3. Perioperative myocardial infarction (MI) was significantly higher (*p* = 0.048) in the obese group (*n* = 25 (6.8%) compared to the non-obese group (*n* = 40 (4.2%) before PSM. However, no significant differences (*p* = 0.299) regarding perioperative MI were observed between both groups after PSM (Figure 2). All-cause in-hospital mortality did not significantly differ between both groups before PSM (*p* = 0.566) and after PSM (*p* = 0.780). No significant differences regarding further postoperative parameters were observed between the unadjusted and the adjusted group.

### 3.4. Primary and Secondary Outcomes by Obesity Classes (I, II, III) before and after PSM

**Figure 3:**
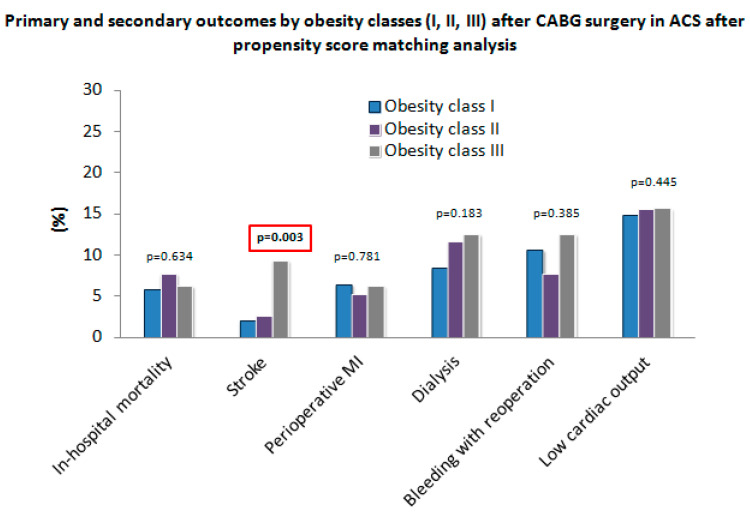
Primary and secondary outcomes by obesity classes (I, II, III) after CABG surgery in ACS after PSM.

All relevant postoperative results regarding obesity classes (I, II, III) before (*n* = 379) and after (*n* = 372) PSM are shown in Table 4. Stroke differed significantly within all three obesity groups before (*p* = 0.018) and after PSM (*p* = 0.003) (Figure 3). No significant differences regarding perioperative MI, respiratory failure, LCOS, dialysis, bleeding requiring re-operation, and all cause in-hospital mortality were observed between the unadjusted and the adjusted group.

## 4. Discussion

We compared non-obese and obese patients regarding their short-term outcomes after bypass surgery in ACS. Both groups showed similar perioperative data. Our study shows that obese patients experienced similar all-cause in-hospital mortality as the non-obese patients (*p* = 0.780).

The evidence regarding the impact of obesity on postoperative outcomes is inconsistent [6,8,15]. Some studies showed that obesity might increase the risk of early adverse results in patients after CABG procedure [2,6,11,13,21,22,23]. Comorbidities, such as diabetes, arterial hypertension, and impaired renal and respiratory function often found in obese patients could affect the results [21,23,24]. Moreover, additional authors reported that obese patients had inferior short-term outcomes following bypass surgery [14,23,24,25]. In contrast, several other studies demonstrated that obesity might be associated with lower operative mortality in patients following a CABG procedure [3,7,10,24,26]. Previous findings motivated us to investigate the impact of obesity on early outcomes in patients who underwent bypass surgery in our study group.

The non-obese group in our trial was not significantly older (*p* = 0.724) compared to the obese group. Nonetheless, obese patients had significantly higher prevalence of diabetes (*p* = 0.004), hyperlipidemia (*p* = 0.004) and arterial hypertension (*p* < 0.001). However, short-term follow-ups failed to show a negative impact of obesity (*p* = 0.780) on all-cause in-hospital mortality.

Our results are consistent with other findings in the literature [9,16,20,24,27,28]. Akinnusi et al. [29] stated that obesity was not associated with higher ICU mortality after a CABG procedure. Moreover, they found no significantly longer duration of mechanical ventilation or ICU length of stay between both groups [29]. Oliveros et al. [30] stated that obese patients experienced lower in-hospital mortality compared to non-obese patients. Furthermore, the ICU-length of stay was significantly longer for overweight patients [30]. Further studies showed significant differences according to in-hospital mortality between obese and non-obese group following bypass surgery [5,31,32]. This mismatch could be explained by various definitions of obesity, patient selection criteria, and different follow-up time [11,33].

Short- and long-term outcomes after STEMI compared to NSTEMI in obese patients remain unclear [34,35,36,37]. Polonski et al. [34] compared treatments and the two-year outcomes in patients with myocardial infarction (MI) (*n* = 8250 with STEMI vs. *n* = 5191 with NSTEMI). The NSTEMI group showed a significantly higher (*p* < 0.001) mortality rate compared to the STEMI group [34]. Moreover, the STEMI group showed a significantly lower (*p* < 0.001) re-infarction rate, and a significantly higher (*p* < 0.001) rate of percutaneous coronary interventions (PCI). 

However, after adjustment for the baseline characteristics and treatment strategy, the NSTEMI group showed a better prognosis in the long-term follow up compared to the STEMI group [34]. Furthermore, Abbot et al. [38] analyzed 1486 patients (*n* = 903 with STEMI vs. *n* = 583 with NSTEMI) after acute MI. The mortality rate was significantly higher (*p* = 0.004) in patients with STEMI compared to patients with NSTEMI. Moreover, authors hypothesized that STEMI might be an independent predictor of mortality [38].

We found no correlation between obesity and an increased duration of mechanical ventilation. Obese patients tend to experience atelectasis, pneumonia, and aspiration during mechanical ventilation [5,39,40]. All of these factors could lead to an increased duration of mechanical ventilation. Moreover, the compliance of the chest wall is reduced in obese patients compared with non-obese patients. Most studies did not present risk-adjusted analyses of the association of BMI and duration of mechanical ventilation [5,31,39,40,41].

The association between obesity and ischemic stroke has been controversially discussed [42,43]. Various authors reported that obesity is a significant risk factor for ischemic stroke [44,45]. Moreover, it could intensify brain injury and might be associated with poor neurological outcomes [45]. In contrast, Kumral et al. [43] reported that obesity was not associated with higher mortality after a 5-year follow-up. Moreover, several studies showed better functional outcomes after stroke in obese patients compared with normal weight patients [46,47]. Authors used the term “obesity paradox” for describing unexpected improved outcomes in obese patients [46]. Further studies hypothesized that ‘’protective’’ peripheral body fat might reduce the inflammatory response and improve outcomes [48,49].

Differences in procedural techniques, surgeon and hospital experience, patient selection and perioperative care should also be taken into account when analyzing the data from our study.

## 5. Conclusions

Our results showed that obesity did not predict increased all-cause in-hospital mortality in patients undergoing CABG procedure in ACS. Moreover, obesity did not significantly increase the risk of other outcomes during the short-term follow-up. However, obese patients have an increased number of risk factors for coronary artery disease compared to non-obese patients. Therefore, emergent CABG was a safe procedure in overweighed patients.

## 6. Study Limitations

This study has several limitations. First, it was a retrospective, single-center analysis, potentially leading to low statistical power. Moreover, we focused on short-term outcomes and did not evaluate long-term results or quality of life measures. Furthermore, data collection was restricted to the available variables in electronic or written patient notes and flowcharts. In addition, CABG surgery was performed by different surgeons, which could lead to a possible bias in the results presented. Investigation of specific pathophysiological conditions was not a part of our study. Therefore, potential explanations of our findings are not feasible from our analysis.

## Figures and Tables

**Table 1:** Patient’s baseline preoperative demographics (*n* = 1346 before PSM and *n* = 744 after PSM).

	Before PSM			After PSM		
	Non-Obese (*n* = 967)	Obese (*n* = 379)	*p*-Value	Non-Obese (*n* = 372)	Obese (*n* = 372)	*p*-Value
Age (years), mean ± SD	67 ± 10.7	66 ± 10.4	0.819	67 ± 10.7	66 ± 10.4	0.724
Female gender, *n* (%)	199 (20.6%)	94 (24.9%)	0.087	81 (21.8%)	94 (25.3%)	0.261
LV-EF (%), mean ± SD	49 ± 15	51 ± 15	0.583	48 ± 14	51 ± 15	0.188
Triple-vessel disease, *n* (%)	780 (80.8%)	311 (82.1%)	0.775	306 (82.5%)	304 (81.7%)	0.787
Unstable angina pectoris, *n* (%)	119 (12.6%)	24 (13.8%)	0.575	47 (12.6%)	52 (14.0%)	0.579
NSTEMI, *n* (%)	532 (55.1%)	245 (64.6%)	<0.001	229 (61.6%)	241 (64.8%)	0.362
STEMI, *n* (%)	316 (32.7%)	82 (21.7%)	<0.001	98 (26.3%)	79 (21.2%)	0.102
Cardiogenic shock, *n* (%)	167 (17.3%)	42 (11.1%)	0.004	52 (14.0%)	40 (10.8%)	0.181
Recent myocardial infarction, *n* (%)	165 (17.1%)	78 (20.6%)	0.132	73 (19.7%)	76 (20.4%)	0.812
Previous PTCA, *n* (%)	210 (21.8%)	70 (18.5%)	0.722	83 (22.4%)	67 (18.0%)	0.134
Previous CABG, *n* (%)	9 (0.9%)	5 (1.3%)	0.534	4 (1.1%)	5 (1.3%)	0.502
Previous stroke, *n* (%)	77 (8.0%)	32 (8.5%)	0.769	35 (9.4%)	32 (8.6%)	0.692
Diabetes, *n* (%)	279 (28.9%)	179 (47.7%)	<0.001	135 (36.2%)	179 (48.1%)	0.004
Hyperlipidaemia, *n* (%)	484 (50.2%)	292 (83.1%)	<0.001	214 (57.5%)	250 (67.2%)	0.004
Smoker, *n* (%)	407 (42.2%)	165 (43.7%)	0.634	165 (44.7%)	161 (43.4%)	0.718
Hypertension, *n* (%)	624 (83.2%)	215 (94.2%)	0.453	319 (85.8%)	350 (94.1%)	0.546
COPD, *n* (%)	101 (10.5%)	49 (13.0%)	0.189	43 (11.6%)	47 (12.7%)	0.653
Atrial fibrillation, *n* (%)	64 (6.6%)	18 (4.8%)	0.199	25 (6.7%)	18 (4.9%)	0.271
Dialysis, *n* (%)	14 (1.4%)	5 (1.3)	0.868	5 (1.3%)	5 (1.3%)	0.622
Renal insufficiency, *n* (%)	117 (12.1%)	46 (12.2%)	0.979	60 (16.2%)	45 (12.1%)	0.107

LV-EF, left ventricular ejection fraction; BMI, body mass index; NYHA, New York Heart Association; COPD, chronic obstructive pulmonary disease; STEMI, ST-Elevation Myocardial Infarction; NSTEMI, Non-ST-Elevation Myocardial Infarction; PSM, propensity score matching; PTCA, percutaneous transluminal coronary angioplasty; CABG, coronary artery bypass grafting surgery.

**Table 2:** Intraoperative data (*n* = 1346 before PSM and *n* = 744 after PSM).

	Before PSM			After PSM		
	Non-Obese (*n* = 967)	Obese (*n* = 379)	*p*-Value	Non-Obese (*n* = 372)	Obese (*n* = 372)	*p*-Value
On-Pump, *n* (%)	877 (90.6%)	300 (85.4%)	0.252	328 (88.9%)	301 (82.7%)	0.065
Off-Pump, *n* (%)	90 (9.4%)	51 (14.6%)	0.129	44 (11.1%)	71 (17.3%)	0.087
Use of 2 ITA grafts, *n* (%)	52 (5.4%)	22 (5.9%)	0.697	16 (4.3%)	22 (6.0%)	0.297
Use of left ITA graft, *n* (%)	732 (94.3%)	262 (94.6%)	0.828	350 (94.9%)	345 (94.8%)	0.966
Use of right ITA graft, *n* (%)	52 (5.4%)	22 (5.9%)	0.697	43 (11.7%)	29 (8.0%)	0.094
Use of radial artery graft, *n* (%)	14 (1.5%)	4 (1.1%)	0.598	7 (1.9%)	4 (1.1%)	0.374
ECMO intraoperatively, *n* (%)	19 (1.9%)	11 (3.1%)	0.241	7 (1.9%)	14 (3.9%)	0.114
IABP intraoperative, *n* (%)	175 (18.2%)	62 (16.8%)	0.345	59 (16.0%)	59 (16.3%)	0.508
CPB time (min), mean ± SD	91 ± 38	96 ± 45	0.084	91 ± 36	95 ± 44	0.254
Cross clamp time (min), mean ± SD	48 ± 20	50 ± 23	0.235	48 ± 21	49 ± 22	0.863
Reperfusion time (min), mean ± SD	33 ± 21	33 ± 24	0.746	32 ± 18	33 ± 23	0.705

CPB, cardiopulmonary bypass; ECMO, extracorporeal membrane oxygenation; IABP, intra-aortic balloon pump; ITA, internal thoracic artery.

**Table 3:** Postoperative data (*n* = 1346 before PSM and *n* = 744 after PSM).

	Before PSM			After PSM		
	Non-Obese (*n* = 967)	Obese (*n* = 379)	*p*-Value	Non-Obese (*n* = 372)	Obese (*n* = 372)	*p*-Value
TIA, *n* (%)	83 (8.9%)	25 (6.9%)	0.077	28 (7.8%)	24 (6.7%)	0.284
Stroke, *n* (%)	35 (3.7%)	12 (3.3%)	0.737	16 (4.4%)	10 (2.8%)	0.254
Perioperative myocardial infarction, *n* (%)	40 (4.2%)	25 (6.8%)	0.048	17 (4.6%)	23 (6.4%)	0.299
CK-MB, 72 h, U/L, mean ± SD	157 ± 144	145 ± 157	0.669	133 ± 121	145 ± 157	0.440
Duration of mechanical ventilation, hours, mean ± SD	18 ± 12	23 ± 13	0.088	19 ± 12	23 ± 14	0.096
Respiratory failure, *n* (%)	20 (2.6%)	10 (3.5%)	0.558	3 (0.8%)	4 (1.0%)	0.546
LCOS, *n* (%)	144 (15.2%)	58 (15.8%)	0.762	45 (12.3%)	56 (15.6%)	0.204
Lactate 72 h, mmol/L, mean ± SD	6.9 ± 4.9	4.9 ± 6.2	0.964	6.4 ± 5.0	4.9 ± 6.2	0.885
Dialysis, *n* (%)	98 (10.3%)	37 (10.1%)	0.935	39 (10.7%)	35 (9.7%)	0.678
Bleeding requiring reoperation, *n* (%)	82 (8.6%)	40 (11.0%)	0.183	27 (7.4%)	38 (10.6%)	0.131
LV-EF (%), mean ± SD	45 ± 14	43 ± 15	0.789	45 ± 15	44 ± 15	0.273
ICU stay, days, median	3 ± 7	4 ± 6	0.306	3 ± 6	4 ± 6	0.538
Hospital stay, days, median	10 ± 13	11 ± 9	0.795	10 ± 7	11 ± 9	0.131
All-cause in-hospital mortality, *n* (%)	54 (5.5%)	24 (6.3%)	0.566	24 (6.4%)	23 (6.1%)	0.780

TIA, transient ischaemic attack; ICU, intensive care unit; CK-MB, creatine kinase-MB; LV-EF, left ventricular ejection fraction; LCOS, low cardiac output syndrome.

**Table 4:** Primary and secondary outcomes by obesity classes (I, II, III).

	Before PSM Obesity Class (*n* = 379)				After PSM Obesity Class (372)			
	I (*n* = 275)	II (*n* = 76)	III (*n* = 28)	*p*-Value	I (*n* = 263)	II (*n* = 77)	III (*n* = 32)	*p*-Value
Stroke, *n* (%)	6 (2.2%)	3 (3.9%)	3 (10.7%)	0.018	5 (1.9%)	2 (2.5%)	3 (9.3%)	0.003
Perioperative myocardial infarction, *n* (%)	19 (6.9%)	4 (5.2%)	2 (7.1%)	0.839	17 (6.4%)	4 (5.1%)	2 (6.2%)	0.781
Respiratory failure, *n* (%)	18 (6.5%)	7 (9.2%)	2 (7.1%)	0.655	18 (6.8%)	7 (9.0%)	2 (6.2%)	0.602
LCOS, *n* (%)	40 (14.5%)	13 (17.1%)	5 (17.8%)	0.593	39 (14.8%)	12 (15.5%)	5 (15.6%)	0.445
Dialysis, *n* (%)	23 (8.3%)	10 (13.1%)	4 (14.2%)	0.233	22 (8.3%)	9 (11.6%)	4 (12.5%)	0.183
Bleeding requiring reoperation, *n* (%)	29 (10.5%)	7 (9.2%)	4 (14.2%)	0.623	28 (10.6%)	6 (7.7%)	4 (12.5%)	0.385
All-cause in-hospital mortality, *n* (%)	16 (5.8%)	6 (7.8%)	2 (7.1%)	0.206	15 (5.7%)	6 (7.7%)	2 (6.2%)	0.634

LCOS, low cardiac output syndrome.

## Data Availability

Data can be obtained on a reasonable request.
